# Health Facility Utilisation Changes during the Introduction of Community Case Management of Malaria in South Western Uganda: An Interrupted Time Series Approach

**DOI:** 10.1371/journal.pone.0137448

**Published:** 2015-09-10

**Authors:** Sham Lal, Richard Ndyomugenyi, Neal D. Alexander, Mylene Lagarde, Lucy Paintain, Pascal Magnussen, Daniel Chandramohan, Siân E. Clarke

**Affiliations:** 1 Department of Disease Control, Faculty of Infectious Tropical Diseases, London School of Hygiene and Tropical Medicine, London, United Kingdom; 2 C/O Vector Control Division, Ministry of Health, Kampala, Uganda; 3 MRC Tropical Epidemiology Group, Department of Infectious Disease Epidemiology, Faculty of Epidemiology and Population Health, London School of Hygiene and Tropical Medicine, London, United Kingdom; 4 Department of Global Health and Development, London School of Hygiene and Tropical Medicine, London, United Kingdom; 5 Institute of International Health, Immunology and Microbiology & Institute of Veterinary Disease Biology, Faculty of Health and Medical Sciences, University of Copenhagen, Denmark; British Columbia Centre for Excellence in HIV/AIDS, CANADA

## Abstract

**Background:**

Malaria endemic countries have scaled-up community health worker (CHW) interventions, to diagnose and treat malaria in communities with limited access to public health systems. The evaluations of these programmes have centred on CHW’s compliance to guidelines, but the broader changes at public health centres including utilisation and diagnoses made, has received limited attention.

**Methods:**

This analysis was conducted during a CHW–intervention for malaria in Rukungiri District, Western Uganda. Outpatient department (OPD) visit data were collected for children under-5 attending three health centres one year before the CHW-intervention started (pre-intervention period) and for 20 months during the intervention (intervention-period). An interrupted time series analysis with segmented regression models was used to compare the trends in malaria, non-malaria and overall OPD visits during the pre-intervention and intervention-period.

**Results:**

The introduction of a CHW-intervention suggested the frequency of diagnoses of diarrhoeal diseases, pneumonia and helminths increased, whilst the frequency of malaria diagnoses declined at health centres. In May 2010 when the intervention began, overall health centre utilisation decreased by 63% compared to the pre-intervention period and the health centres saw 32 fewer overall visits per month compared to the pre-intervention period (p<0.001). Malaria visits also declined shortly after the intervention began and there were 27 fewer visits per month during the intervention-period compared with the pre-intervention period (p<0.05). The declines in overall and malaria visits were sustained for the entire intervention-period. In contrast, there were no observable changes in trends of non-malarial visits between the pre-intervention and intervention-period.

**Conclusions:**

This analysis suggests introducing a CHW-intervention can reduce the number of child malaria visits and change the profile of cases presenting at health centres. The reduction in workload of health workers may allow them to spend more time with patients or undertake additional curative or preventative roles.

## Introduction

Malaria is one of the leading causes of under-5 child morbidity and mortality in low income countries where children have limited access to public healthcare [1]. In order to increase access to prompt effective treatment for malaria, many countries have implemented community health worker (CHW) strategies as part of national malaria control programmes [2]. They have been effective in reducing under-5 mortality by 40% and reducing the incidence of severe malaria by 50% [3–5]. Recently CHWs have been endorsed by the World Health Organization (WHO), United Nations Children’s Fund (UNICEF) and international donors as a strategy to meet the fourth Millennium Development Goal (MDG) of reducing under-5 mortality from 1990 levels by two-thirds by 2015.

In many Sub-Saharan African (SSA) countries without CHW interventions, malaria accounts for an average of 50% of all outpatient department (OPD) visits and between 20% to 90% of all hospital admissions [6–9]. When malaria treatment is available in the community, there may be a shift in treatment seeking away from health centres to CHWs within a child’s community [10]. This may partly arise by the closer proximity of the treatment provider in the child’s area compared to a health centre. Secondly, there may be reduced household costs associated with seeking treatment closer to home and finally, CHWs are often elected by the community and might be more trusted and accepted providers compared to health centre staff. We hypothesise that changes in treatment seeking may affect the conditions that present at health centres. For example, if a large proportion of febrile visits are treated in the community, the proportion of non-malaria fever visits may increase among children attending health centres.

Many SSA countries are scaling up CHW interventions, specifically integrated community case management (iCCM). The primary evaluations of these programmes have been on CHW’s compliance to guidelines and disease-specific impacts whilst the effects of the CHW programmes on public health centres have not been widely documented [11]. The aim of this paper is to examine the effects of introducing a CHW intervention for malaria on health centre utilisation by children under-5 in South Western Uganda.

## Materials and Methods

### Ethical approval

Data analysed comes from routinely available, anonymised health management information systems (HMIS) captured from three public health centres in the study area. Ethical approval for the collection and analysis of the anonymised HMIS data was approved by institutional review boards at the Uganda National Council of Science and Technology, Ministry of Health, Uganda and the London School of Hygiene & Tropical Medicine. Local approval to collect and analyse the HMIS data was also sought from the district health officer and health centre staff.

### Study context

This study was conducted in parallel to an on-going CHW-intervention which examined the impact of malaria rapid diagnostic tests (mRDTs) on the proportion of children under-5 years receiving appropriately targeted artemisinin based combination therapy (ACT) (Nydyomugenyi 2015 in press). The intervention was conducted in a moderate-to-high malaria transmission setting within Bwambara Sub-county, Rukungiri District, South Western Uganda [12]. Bwambara lies 400km west of the capital Kampala with a population of approximately 28,900 with more than 85% living in rural areas [13]. The area experiences a bimodal pattern of annual rainfall with a long rainy season between September and December and a short rainy season from March to May. Malaria transmission is perennial with peaks in incidence shortly after the rains. The public health system has three health centres that serve the sub-county’s population. Two are classified as public health centre II and are limited to outpatient services. There is one health centre III and in addition to outpatient services, it has maternity services, an inpatient ward and microscopy for diagnosing malaria, it also acts as a referral centre for both lower level II health centres (Table 1) [14].

**Table 1:** The structure of the Uganda National Health system in Bwambara Sub-county.

Health Centre	Services	Number of Health Centres	Catchment	Population Served	Method of malaria diagnosis
**Health Centre II**	Outpatient	2	Parish	5,000	mRDT
**Health Centre III**	Outpatient, maternity services, inpatient ward, microscopy	1	Sub-county + Parish	20,000	mRDT + light microscopy

Adapted from Government of Uganda, Health Sector Strategic Plan III, 2010/11–2014/15[14]

#### Community health worker intervention

The primary objective of the CHW-intervention was to evaluate the effectiveness of mRDTs used to diagnose and treat only malaria with ACTs compared with the presumptive diagnosis and treatment of malaria. The intervention was implemented at the village level, where 63 clusters were randomised to either the mRDT diagnosis arm or the presumptive diagnosis arm. In total 189 CHWs were trained (3 per village) between January and February 2010, with separate training sessions for each arm. All CHWs were trained on how to examine the febrile children, take their history and record basic patient details on treatment registers. CHWs were also taught how to recognise children with severe malaria and symptoms of other illnesses and how to refer them to the nearest public health centre for further management. The 31 villages in the mRDT arm received additional training in malaria diagnosis using mRDTs and to only prescribe ACTs after a positive test result (further trial details available at www.actconsortium.org/RDThomemanagement). In February 2010, public meetings were held with participating villages to disseminate key messages that included, a) not all fevers are malaria, b) a diagnostic test was advisable before treatment with an antimalarial and c) tests were available from CHWs in the intervention arm. After CHWs completed training, the intervention was implemented in all villages during the first week of May 2010. Immediately prior to the trial there were no other community case management services operating in the sub-county.

#### Data collection

To analyse the impact of the CHW-intervention on the changes in health centre visits before and after the introduction of the intervention, we reviewed OPD records on children under-5 from the three health centres. Data were extracted from OPD treatment registers maintained by the health centre staff for the 12 months prior to the commencement of the CHW-intervention (May 2009 to April 2010, the “pre-intervention period”) and for the full 20 months of the intervention (May 2010 to December 2011, the “intervention-period”). The intervention did not introduce changes in the practice of OPD data collection or recording at any of the three health centres. The routinely available OPD data captured demographic data on age, sex, village of residence, diagnosis and date of visit. Malaria diagnosis at health centres in Uganda was based on a presumptive diagnosis until 2009, when the National Malaria Control Programme adopted the WHO recommendation of universal parasitological testing at all levels of the health care system [9]. Diagnosis of malaria at the health centres varied by the type of health centre: the primary method of malaria diagnosis at the two level II health centres was mRDT, whilst at the level III health centre the main method of malaria diagnosis was either by light microscopy or mRDTs. When neither of these methods were possible, for example due to a stock-out of mRDTs, a presumptive diagnosis was made. However, the method of diagnosis for malaria or other conditions was not routinely recorded in the registers by health centre staff.

**Fig 1:**
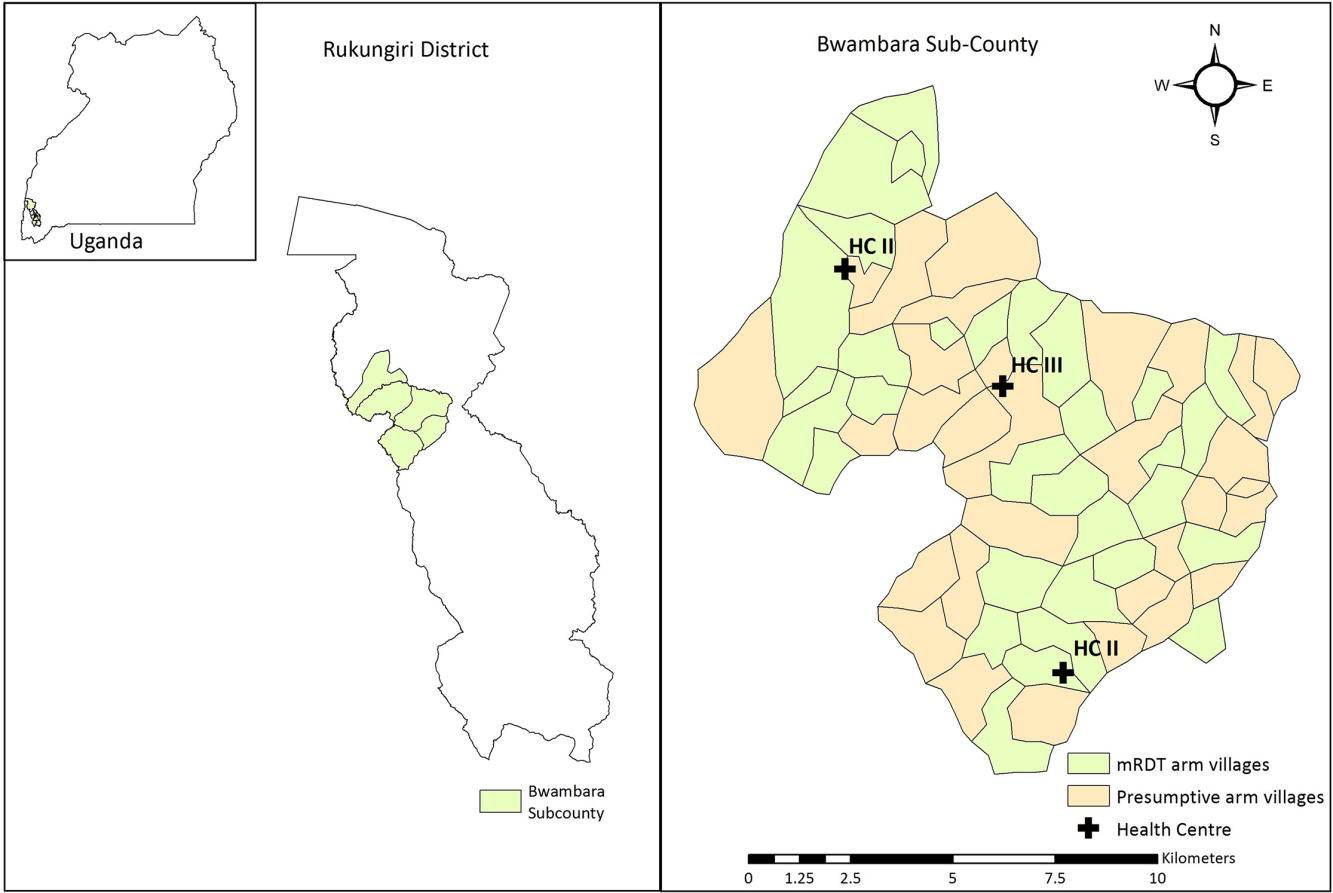
Map of study area Bwambara Sub-county. The Map was generated by authors in Arc GIS 10.3. Sub-county boundaries were sourced from Global Administrative Areas (http://gadm.org)

This analysis of health centre OPD visits was conducted within the context of a cluster randomised trial (CHW-intervention) and the locations of each health centre in relation to the randomised villages (clusters) are shown in Fig 1. The aim of this analysis was to examine the changes in the mean number of visits at health centres when a CHW-intervention for malaria was implemented in the health centre’s catchment area.

#### Statistical methods

The diagnoses recorded in OPD registers by health centre staff were categorised into commonly occurring childhood conditions (malaria, respiratory tract infections (RTIs), pneumonia, diarrhoea, helminths and other infections) during the pre-intervention and intervention-period for each health centre and collectively for all three health centres. Statistical chi-square tests were calculated to test the null hypothesis that there were no differences in the age, sex and diagnoses made between the pre-intervention and intervention-period. In addition, we calculated standardised differences the adjusted residuals (ARs) between the observed and expected frequencies. These were calculated by dividing the residual (observed minus the expected frequency) by the standard error of all the residuals. They measure the number of standard errors the residual falls from zero and follow a standard normal distribution that allows hypothesis testing [15,16].

An interrupted time series (ITS) approach was used to analyse the longitudinal OPD visit data and evaluate the impact of introducing a CHW-intervention on health centre utilisation, taking into account background secular trends [17]. In an ITS approach, each unit of analysis acts as its own control and one compares changes in the outcome of interest (here monthly OPD visits) before and after the start of the CHW-intervention [17]. To undertake the ITS analysis, segmented linear regression was used to estimate the effect of the intervention on three outcomes at the health centres (malaria visits, non-malaria visits and overall visits) before and after the introduction of the CHW-intervention. The specification of the linear regression model to be analysed for each outcome is:

$$Y_{t}=\beta_{0}+\beta_{1}{\times time}_{t}+\beta_{2}{\times intervention}_{t}+\beta_{3}\times {time\;after\;intervention}_{t}+\varepsilon_{t}$$ (1)

Where *Y*_*t*_ denotes the number of child visits (outcome) in month *t* at a health centre for malaria, non-malaria or overall visits; *time* is a continuous variable indicating the time in months at time *t* over the entire period; *trial* is a dichotomous indicator variable for the CHW-intervention, equal to zero before the start of the intervention and equal to one after the start of the intervention; *time after intervention* is a continuous variable counting the number of months after the CHW-intervention starts, and equal to zero before the intervention starts. Finally, *ε*_*t*_ is an error term at time *t* that represents random variability not explained by the model.

**Fig 2:**
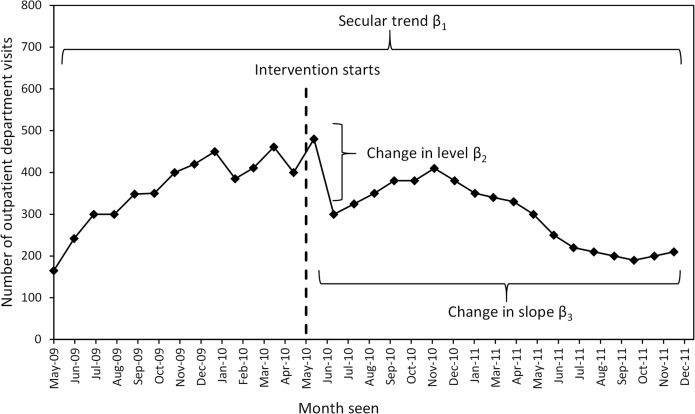
Graphical representation of the parameters in the segmented regression Eq 1 using artificial data.

With the variables defined as above, *β*_0_ estimates the number of visits per month at time 0 (baseline level of visit); *β*_1_ estimates the secular trend in the number of visits per month over the entire period; *β*_2_ estimates the level change in the number of visits per month immediately after the trial (the immediate effect of the intervention on the outcome of interest); *β*_3_ is the coefficient that captures the change in trend in the number of visits after the start of the intervention. The sum of *β*_1_ and *β*_3_ is the intervention-period slope. By controlling for baseline level and trend, the model estimates level and trend changes associated with the start of the intervention. The coefficients are illustrated graphically in Fig 2 and describe the analysis undertaken using Eq 1.

The Durbin-Watson statistic was used to test for the presence of autocorrelation on the error term *ε*_*t*_. When this was detected, regression coefficients were estimated using a Prais-Winsten estimator [18].

Finally, assuming that the changes in trend would remain the same in the future, we used the estimated regression coefficients to predict changes in each outcome at 3, 6, 12 and 18 months after the introduction of the intervention [19]. These estimates were compared with a simulated counterfactual, to project expected values in the absence of the intervention. This assumes the trends continue along the same trajectory as in the pre-intervention period (*β*_3_ = 0). Percentage changes were calculated for any visit and malaria-specific visits for each health centre separately and for all three combined.

Data were entered using Microsoft Access 2007 (Microsoft Inc., Redmond, Washington), cleaned and analysed using STATA version 13.1 and the ‘prais’ package (STATA Corporation, College Station, Texas).

## Results

Throughout the 12-month pre-intervention and 20-month intervention-period, a total of 11,422 visits were reported from all health centres and 97% of these came from the same sub-county where the CHW-intervention was implemented. Approximately 3% of visits originated from neighbouring sub-counties and were excluded from the analysis. The utilisation of all three health centres was higher during the pre-intervention period (509 visits per month) compared to the intervention-period (265 visits per month).

The age distribution of visits at health centres was statistically different between the two periods (χ^2^ = 66.6, df = 4, p<0.001). The ARs revealed the largest changes in age frequencies were in children aged <1 year (AR = 6.7, p<0.001), 1.0–2.9 years (AR = -5.5, p<0.001) and there were no significant changes for children aged between 3.0–4.9 years (AR = 0.2, p<0.195) or 5.0–11.0 years (AR = 0.4, p = 0.363) (Table 2). The change in age frequencies suggests visits of infants (<1 year) increased during the intervention-period compared to the pre-intervention period, whilst there were decreases in visits from children aged between 1.0–4.9 years. There were no significant differences between the frequencies of male or female visits between the two time periods (Table 2).

**Table 2:** Characteristics and diagnoses made of children attending all three health centres.

	12 month Pre-intervention period (%)	20 month Intervention-period (%)	Adjusted Residual	P-value
**Total number of visits**	6110	5312		
***Age in years*** ^*$*^				
**<1.0**	1650 (27.3)	1737 (33.1)	6.7	<0.001
**1.0–2.9**	3252 (53.7)	2548 (48.5)	-5.5	<0.001
**3.0–4.9**	1124 (18.6)	942 (17.9)	-0.9	0.195
**5.0–11.0**	25 (0.4)	24 (0.5)	0.4	0.363
***Sex*** ^*^				
**Male**	2792 (46.2)	2441 (47.1)	-1.0	0.456
**Female**	3257 (53.8)	2740 (52.9)	1.0	0.456
**Total diagnoses made** ^€^	9721	8708		
***Diagnosis*** ^*%*^				
**Malaria**	4400 (45.3)	1845 (21.2)	-37.4	<0.001
**Respiratory tract infection**	2644 (27.2)	2735 (31.4)	6.3	<0.001
**Pneumonia**	59 (0.6)	123 (1.4)	5.5	<0.001
**Diarrhoea**	538 (5.5)	593 (6.8)	3.6	<0.001
**Helminths**	428 (4.4)	771 (8.9)	12.2	<0.001
**Other**	1652 (17.0)	2641 (30.3)	21.4	<0.001
**Average number of diagnoses per visit**	1.5	1.5		

^$^Age missing 59 pre-intervention period, 61 intervention-period

*Sex missing 61 pre-intervention period and 131 intervention-period

€ It was possible for children attending health centres, to have more than one diagnosis

^%^RTI includes cough, cold, flu, excludes pneumonia, TB and asthma. A child can have more than one diagnosis.

Other diagnoses pre-intervention include: Skin infections (1.9%), burns, wounds, injuries (0.3%), eye infections (2.2%), epilepsy (0.2%), ear conditions (1.4%), gastro intestinal infections (0.6%), STIs (0.03%), fungal infections (0.2%), viral infections (0.07%); Other diagnoses trial period include: Skin infections (4.5%), burns, wounds, injuries (0.9%), eye infections (5.3%), epilepsy (0.7%), ear conditions (2.6%), gastro intestinal infections (0.8%), STIs (0.1%), fungal infections (0.7%), viral infections (0.6%)

### OPD Diagnoses

Table 2 also shows the combined childhood diagnoses made by health centre staff at the three health centres. Malaria contributed to approximately 45% of all diagnoses made during the pre-intervention period, followed by RTIs (27%), diarrhoea (6%), helminth infections (4%), pneumonia (1%) and other diagnoses (17%). During the intervention-period the frequencies of diagnoses at the three health centres changed significantly when compared to the pre-intervention period (χ^2^ = 1346.4, df = 4, p<0.001). There was a significant decline in malaria diagnoses observed between the two time periods (AR = -37.4, p<0.001), whilst the frequency of other diagnoses (AR = 21.4, p<0.001), helminths (AR = 12.2, p<0.001), RTIs (AR = 6.3, p<0.001), pneumonia (AR = 5.5, p<0.001) and diarrhoea (AR = 3.6, p<0.001) all increased from the pre-intervention to the intervention-period.

### Trends in malaria, non-malaria and overall visits

**Fig 3:**
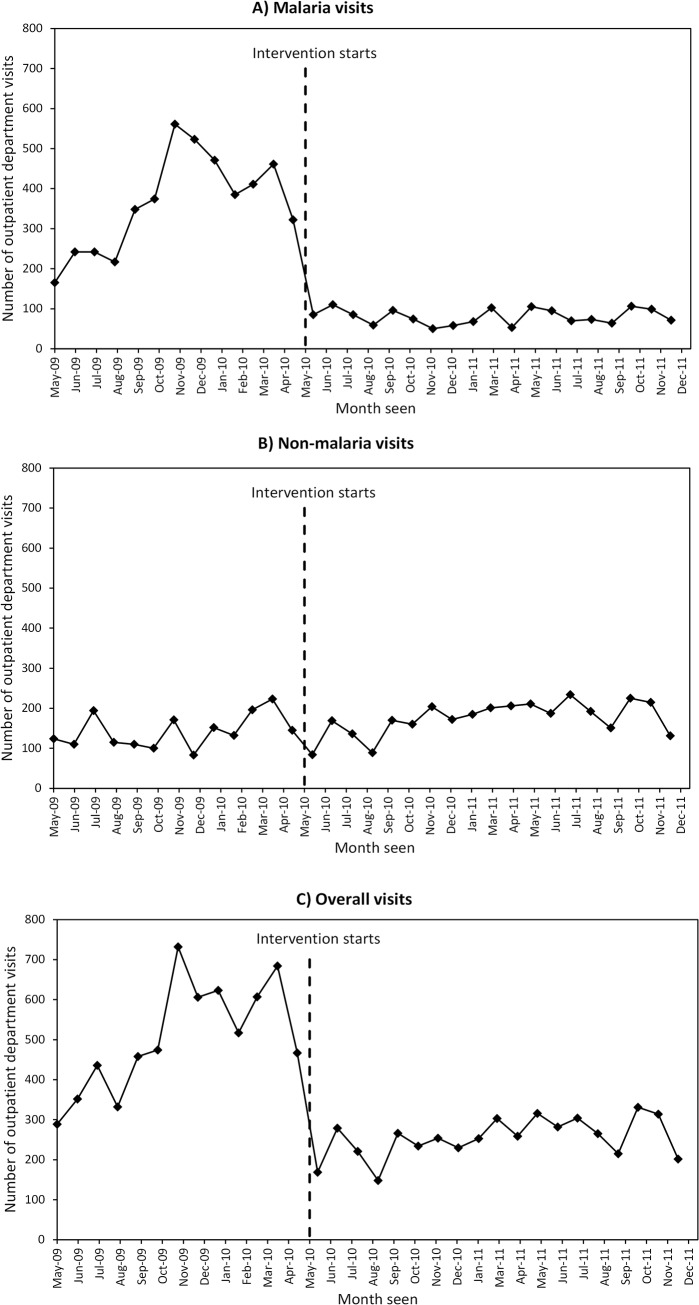
Trends in visits during the pre-intervention and intervention-period at all health centres. A) Malaria visits, B) Non-malaria visits and C) Overall visits.

The graphs in Fig 3 show the trends of a) malaria, b) non-malaria and c) overall visits at the three health centres combined. During the pre-intervention period there were seasonal peaks of malaria visits during the two rainy seasons between October-December 2009 and between March-April 2010. When the intervention started in May 2010, a drop in malaria visits was observed, with no seasonal variation in visits during the intervention-period (Fig 3A). In contrast, the patterns of non-malaria visits did not change substantially between the two periods (Fig 3B). Finally, the trends of overall visits during the pre-intervention and intervention-period are shown in Fig 3C. Collectively, the health centres saw similar patterns of visits as those for malaria, with two large peaks of visits coinciding with the rainy seasons. Shortly after these peaks the health centres saw a sharp drop in visits when the CHW-intervention began and there appeared to be substantially fewer visits during the intervention-period compared with pre-intervention period (Fig 3C).

### Segmented regression results

Table 3 shows the segmented regression results for malaria, non-malaria and overall visits for the three health centres combined. The regression coefficient *β*_1_ estimates the average number of visits during the pre-intervention period and indicates health centres saw an increase of approximately 20 malaria visits per month. The average difference between pre-intervention trend and the intervention-period slope is estimated by *β*_3_ and after the intervention began there were 27 fewer malaria visits per month compared with the pre-intervention period (Table 3). The health centres saw a sharp change in malaria visits in May 2010 within one month after the start of the intervention, and an estimate of the percentage change was calculated using the regression coefficients estimated from Table 3. These suggest that in May 2010 there was an average of 261.7 visits per month, whilst in the absence of the intervention 722.0 malaria visits per month would have been observed. This suggests the absolute effect of the intervention was a sharp drop of 63.8% in malaria visits in May 2010. Despite the clear changes in trends for malaria visits, there was poor evidence to indicate changes in non-malaria visits. The results in Table 3 indicate an increase in the mean number of non-malaria visits during the pre-intervention period and that these declined by 40 visits per month when the intervention started, however these changes did not reach significance.

**Table 3:** Changes in level and trend of malaria, non-malaria and overall visits at three health centres combined, results from a segmented linear regression model, unadjusted and adjusted results.

	Unadjusted			Adjusted^^^		
	Malaria visits^$^	Non-malaria visits	Overall visits	Malaria visits^$^	Non-malaria visits	Overall visits
Constant (*β*_0_)	207.7**	106.0***	296.3***	204.0**	106.3***	294.4***
	(70.9)	(23.3)	(46.5)	(70.8)	(23.7)	(45.5)
Secular trend (*β*_1_)	19.2*	5.6	32.7***	15.7	6.0	29.8***
	(8.9)	(3.2)	(6.3)	(8.9)	(3.4)	(6.5)
Change in level after intervention starts (*β*_2_)	-245.0***	-39.8	-427.9***	-218.7**	-42.6	-408.3***
	(65.9)	(27.1)	(54.0)	(62.9)	(28.3)	(54.3)
Change in slope after intervention starts (*β*_3_)	-27.1*	-1.8	-32.3***	-24.7*	-2.1	-30.0***
	(11.0)	(3.5)	(7.0)	(11.1)	(3.6)	(7.0)
Rainy season (*β*_4_)				51.8*	-6.1	42.8
				(23.7)	(14.6)	(28.1)

Standard errors in parenthesis

*p<0.05

** p<0.01

*** p<0.001

^$^model includes an adjustment for autocorrelation

^adjusted for bimodal rainy seasons.

The results of the segmented regression analysis for overall visits in Table 3 reflect similar findings as those presented in Fig 3C. At the beginning of the period of observation (May 2009) 296 consultations were reported from all three health centres and during the pre-intervention period the average number of visits increased by 33 per month. These increases at health centres were reversed with a drop shortly after the start of the intervention with 428 fewer monthly visits compared to the pre-intervention period, which represents a 63% decrease in the mean number of overall visits at health centres between the two time periods (p<0.001). The average difference between pre-intervention trend and the intervention-period slope found that health centres received 32 fewer visits per month compared with the pre-intervention period (Table 3). The number of visits during the intervention-period remained constant throughout the period because there was no change in trend during the intervention-period (Fig 3C).

The adjusted segmented regression models incorporated an indicator variable to capture the effects of the rainy seasons on visits (Table 3). When compared to the unadjusted results, there was no longer evidence for an increasing trend in malaria visits at health centres during the pre-intervention period, and the magnitude of decline in malaria visits when the intervention began was slightly smaller (219 visits per months) compared with the unadjusted results (245 visits per month). In addition, malaria visits peaked by approximately 52 visits per month during the rainy seasons. There were no substantial changes in non-malaria visits and overall visits between the adjusted and unadjusted results (Table 3).

Table 4 uses the coefficients presented in Table 3 to calculate the simulated counterfactual visits that would have occurred in the absence of the CHW-intervention. At all the health centres these were calculated for malaria, non-malaria and overall visits at 3, 6, 12 and 18 months after the start of the intervention. Three months after the intervention began malaria and overall visits declined by 68% and continued to decline throughout the course of the intervention and there were no significant changes in non-malaria visits.

**Table 4:** Percentage change in health centre visits for malaria, non-malaria visits and overall visits over time unadjusted and adjusted results.

	Unadjusted			Adjusted^^^		
	Malaria visits^$^	Non-malaria visits^*^	Overall visits	Malaria visits	Non-malaria visits^*^	Overall visits^*^
% Change 3 months after intervention starts	-68.6	-24.1	-67.9	-69.7	-25.2	-68.6
% Change 6 months after intervention starts	-75.8	-24.7	-71.2	-77.9	-26.1	-72.0
% Change 12 months after intervention starts	-86.8	-25.8	-76.1	-90.4	-27.4	-77.0
% Change 18 months after intervention starts	-94.5	-26.5	-79.5	-99.5	-28.4	-80.5

^$^Model includes an adjustment for autocorrelation

*Percentage changes not significantly different from zero

^adjusted for bimodal rainy seasons.

**Fig 4:**
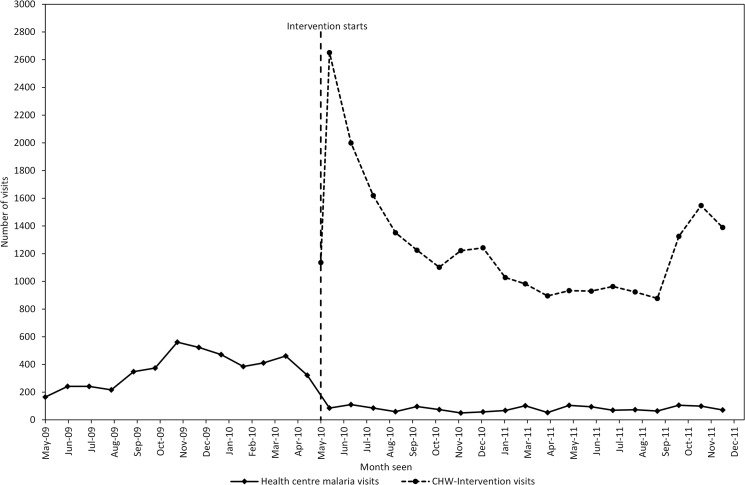
Trends in malaria visits at health centres and visits to the community health worker intervention, during the pre-intervention and intervention-period.

The declines in overall health centre are plotted alongside all visits to CHWs as part of the intervention. The data in Fig 4 shows when the intervention began in May 2010 there was a sharp rise in visits at CHWs which gradually declined in subsequent months, except for a peak in visits from October to December 2011. The average number of visits at the CHW-intervention was 1,267 per month, which exceeded the average number of malaria visits at health centres (80 per month) during the months of the intervention-period.

### Supplementary analyses

An additional analysis was undertaken to compare the age distributions and diagnoses between two equal time periods, a 12-month pre-intervention period and a 12-month intervention-period (Table A in S1 File). This found the same changes in age visits as the analysis with a 20-month intervention-period. Children <1 year increased during the 12-month intervention-period, whilst children aged between 1.0–2.9 years decreased and no significant changes were seen with children aged 3 years or greater (Table A in S1 File).The analysis of diagnoses found similar changes, as the frequency of malaria diagnoses during the 12-month intervention-period decreased, RTIs, pneumonia, diarrhoea, helminths and other diagnoses all increased during the intervention-period (Table A in S1 File).

The aggregated results mask some of the heterogeneity seen at the individual health centres, and to examine patterns of visits at each individual health centre a further set of analyses were undertaken (Table B in S1 File). During the pre-intervention period, level II health centres saw a monthly increase in malaria and overall visits, ranging between 9 and 18 visits per month. One month after the start of the intervention, both level II health centres saw a drop in visits that ranged from 127 to 142 fewer visits per month (Table B in S1 File). Shortly after the drop, in the intervention-period health centres IIs saw between 8 and 20 fewer visits per month compared to the pre-intervention period. Despite the changes seen at each individual health centre for malaria and overall visits, there were no obvious changes at each health centre for non-malaria visits (Table C in S1 File). The higher level health centre III saw no significant change in the number of monthly malaria or overall visits during the pre-intervention period, however there was a significant drop in overall visits when the intervention began (Table F in S1 File).

The regression results adjusted for the rainy seasons were broadly similar compared to the unadjusted results for each health centre. However, health centre III saw a large reduction of 129.2 malaria visits compared with a non-significant reduction of 44.2 visits in the unadjusted model (Table B in S1 File). The adjusted results also showed an increase of between 15–18 malaria and overall visits per month at Kikongi Health Centre II during the rainy seasons (Table B in S1 File, Table F in S1 File).

The percentage change in visits at each health centre was also calculated using a simulated counterfactual. Both health centre IIs saw an estimated 90% decline in malaria visits and both centres followed similar patterns of decline 18 months after the intervention started (Table C in S1 File). There was little difference between the adjusted and unadjusted percentage changes, suggesting the rainy season did not affect the results.

## Discussion

This interrupted time series analysis shows a decline in health centre utilisation for malaria and overall visits shortly after the introduction of a CHW-intervention for malaria diagnosis and treatment. This decline did not occur gradually over the study period, rather there was a clear drop in visits that coincided with the introduction of the intervention to the surrounding communities. Three months after the invention began malaria and overall visits declined by an average of 70% across the three health centres, however there was little evidence to suggest any change in the average number of non-malaria visits at the health centres. The reductions in both malaria visits and overall visits were sustained beyond an initial three-month period, after 18 months malaria visits had decreased by 95% and overall visits by 80% when compared to the pre-intervention period. In relation to these changes a marked increase in utilisation of CHWs (1,267 visits per month) was reported, when compared with malaria-visits at health centres (Fig 4). This suggests there was a shift in caretaker treatment seeking from public health centres to CHWs during the intervention-period and that access to malaria diagnosis and treatment also increased when compared to the pre-intervention period. Both the shift in treatment seeking and the increased access suggest a greater utilisation of case management services for those in rural areas. These findings are similar to household cross-sectional surveys where 27%-59% of caretakers first sought treatment from CHWs treating malaria [20–22].

In this study, CHWs only treated malaria and saw the proportion of malaria diagnoses approximately halved at health centres between the pre-intervention and intervention-periods. The diagnoses made for RTIs, pneumonia, diarrhoea, helminth and other diagnoses all increased during the intervention-period. There could be a number of explanations for this change. Firstly, there were more overall visits to CHWs by caretakers and a larger number of non-malaria visits may have been referred to health centres, which would have been unlikely in the absence of a CHW-intervention. Secondly, the change in diagnoses could be due to health workers being more alert and aware to the possibility of other diagnoses, knowing that CHWs in their health centre catchment area are diagnosing and treating malaria, and therefore tend to diagnose other conditions over malaria. However, previous studies report a low frequency of referral making by CHWs and a low compliance to referral advice by caretakers [23,24]. This was also confirmed in this study by examining CHW’s and health centre worker’s treatment registers from the intervention trial, which suggested that only 13% of children referred from a CHW to a health centre actually went (i.e. completed the referral). Thus referral may explain only a small fraction of visits attending for non-malaria diagnoses, whilst the change in clinical decision-making by health workers may be an explanation that accounts for the increase in the diagnoses of non-malarial conditions.

There are a number of limitations that need to be considered when interpreting the findings from this analysis. The impacts of new healthcare programmes are ideally evaluated using experimental study designs such as cluster randomised controlled trials. However, when programmes begin to scale up at a district or national level they are not randomised and evaluating impacts on health systems can be achieved using routinely available health centre data. Therefore a non-experimental interrupted time series approach was used with segmented regression models and these are becoming more commonly used to assess impacts of health programmes or policies [18,25]. Another approach to analyse the routinely available visit data would be to use auto-regressive integrated moving average modelling (ARIMA) [26]. However, ARIMA modelling-based approaches require a large number of data points over time and much of the routinely available data from low and middle-income countries are not available for long periods of time and can be irregularly reported. As with all non-randomised studies, causal inference from the interrupted time-series approach is limited because it is impossible to rule out alternative explanations for observed changes in visits at health centres. For example, one explanation for the observations found could be a change in data recording practices, however the study did not introduce data collection tools at the health centres to systematically collect data over the entire period of the analysis and no other changes in routine reporting took place during the period of this analysis. Another explanation for the decrease in malaria visits could be the misclassification of diagnoses as malaria instead of other non-malaria febrile illness. During the pre-intervention period if health centres experienced stock-outs of mRDTs and continued with a presumptive diagnosis of malaria this may have resulted in a practice of over diagnosing visits as malaria. In contrast, during the intervention-period health centres were supported by the project with regular supplies of mRDTs, and the increased stock and use of mRDTs during this period may have improved the accuracy of malaria diagnoses compared to the pre-intervention period.

Another explanation for the changes in utilisations observed between the two periods could be the changes in individual demographic or socio-economic characteristics of caretakers visiting health centres. These may have changed between the pre-intervention period and the intervention-period and may have led to different patterns of health centre utilisation. However, as the unit of analysis in the segmented regression model was the monthly number of visits, it was not possible to undertake an analysis with individual-level covariates nor was it possible to determine whether there was a change in characteristics visiting between the two periods. Regardless, routine OPD data does not include detailed characteristics or socio-economic information on each visit. Individual level characteristics, such as child age or sex, would only confound the time series results if they were associated with the outcome and changed in relation to the timing of the intervention.

Another set of factors to be considered is whether malaria risk declined over the time period of this study. Rainfall is a predictor of malaria admissions to hospitals and may have changed between the pre-intervention and intervention-period [27]. Rainfall affects vector density by providing breeding sites for vectors and their development during the immature stages of the mosquito [28]. We estimated monthly rainfall in South-Western Uganda from the National Oceanic and Atmospheric Administration satellite, and did not change between the pre-intervention and intervention-period (32mm and 31mm, respectively), suggesting it is unlikely rainfall affected the changes in health centre visits found in this analysis [29]. Similarly parasitological malaria surveillance data collected from a neighbouring district suggested prevalence was comparable during pre-intervention period (43.4%) and intervention-period (43.9%) [12]. Finally, during the intervention-period CHWs were also mobilised to distribute insecticide-treated nets (ITNs) to all villages and other studies have found net distribution may play a role in decreased utilisation of health services for malaria [30]. Four months after the start of the CHW-intervention in May 2010 ITNs were distributed to all villages. Therefore, ITN distribution could not have explained the immediate decline in malaria visits the subsequent declines 3 months after the intervention. However, it is plausible that ITNs may have supported the continued reductions in malaria trends during the intervention-period in parallel with the CHW-intervention. Unfortunately it is not possible to separate effects of ITNs and the CHW-intervention on health centre visits in this study. Another explanation for the abrupt decrease in visits during the intervention-period could be a sharp decline in the population under-5 years within the sub-country. However, this seems unlikely and was not reported by the project or health staff.

Despite these potential limitations and the relatively short period of observation, it is unlikely that the sharp declines in malaria and overall visits observed would be seen in a short time frame in the absence of our CHW-intervention. We are thus confident that the changes we observed can be attributed to the intervention operating within the population catchment area of the three health centres. This is further supported by the timing of the dramatic decreases in malaria and overall visits reported by health centres that coincided with the same month as the start of the intervention. Data from the CHW treatment registers suggest that all CHWs in the sub-county were reporting management of malaria visits in the first month of the intervention.

These findings are consistent with all previous observational studies that have documented the effects of health centre utilisation during the introduction of a community case management programme. Studies in Burkina Faso, Rwanda and Southern Tigray, Ethiopia reported reductions in overall visits to public health centres of 83%, 48%, and 46% respectively [10,31,32]. It is important to note that methodological differences between these studies, in terms of study design, length of the CHW-intervention, case definitions for malaria and multiple CHW activities, restrict cross country comparisons. The study in Rwanda by *Sievers et al* was the only study to capture changes in diagnoses, finding a 24 percentage point increase in non-malaria admissions between the pre and post-intervention period [31]. However, ITNs were also distributed in addition to training CHWs to diagnose malaria presumptively, therefore it is not possible to discern the relative contribution of the two control activities to the reduction in admissions in Rwanda [31].

In SSA countries where malaria poses a considerable burden on the health system and where there is a shortage of healthcare workers, our analysis suggests CHW-interventions can alleviate the often overwhelming caseload at health centres. Many of these countries, including Uganda face a shortage of qualified healthcare workers and WHO recommends a ratio of 2.3 healthcare workers per 1,000 population as a minimum to meet the MDGs and Uganda’s ratio remains below the target level at 1.8 per 1,000 [33–35]. A low healthcare worker to patient ratio results in greater caseload and poorer quality of care due to time pressures that affect their ability to follow guidelines and best practices, which in turn results in poor patient outcomes such as readmissions and increased mortality [36]. From our study, we found visits can be reduced by 63% immediately after the introduction of a CHW-intervention and the reduction can be sustained throughout the duration of the intervention. Shifting workload from health workers to CHWs suggests more consulting time would be available for patients at health centres, which has shown to improve patient diagnoses and treatment in low and middle income countries [37,38]. It may also allow the role of health workers to be expanded to include other ameliorative tasks, outreach services or the supervision of CHWs.

## Conclusion

The introduction of CHWs can reduce the number of patient visits presenting as malaria and change the profile of cases seen at health facilities. As malaria endemic countries begin to scale up community-based treatment interventions such as iCCM, there may be reductions in the utilisation of health services as caretakers use CHWs instead. There is a need for further research to document the impact these changes may have on health services and healthcare financing. Priorities for future research should include estimating changes in caretaker’s treatment seeking behaviour, allocation of health worker’s time, quality of care and the diagnoses being made at health centres when CHWs are implemented. This evidence would help plan how available healthcare resources can be distributed more efficiently in countries with high patient caseloads and constrained healthcare budgets.

## Supporting Information

S1 File Twelve month intervention-period analysis and analysis by each health centre. (DOCX)
