# Supplementary material for: Health Facility Utilisation Changes during the Introduction of Community Case Management of Malaria in South Western Uganda: An Interrupted Time Series Approach
Source: PLoS One. 2015 Sep 10;10(9):e0137448. doi: 10.1371/journal.pone.0137448 (PMC4565684; doi:10.1371/journal.pone.0137448)
Supplement: S1 File — (DOCX) [file pone.0137448.s001.docx]

|  | 12 month  Pre-intervention period (%) | 12 month  Intervention-period (%) | Adjusted Residual | P-value |
| --- | --- | --- | --- | --- |
|  |  |  |  |  |
| Total number of visits | 6110 | 3083 |  |  |
| *Age in years^$^* |  |  |  |  |
| <1.0 | 1650 (27.3) | 998 (32.8) | 5.4 | <0.001 |
| 1.0 – 2.9 | 3252 (53.7) | 1495 (49.2) | -4.2 | <0.001 |
| 3.0 – 4.9 | 1124 (18.6) | 541 (17.8) | -1.0 | 0.171 |
| 5.0 – 11.0 | 25 (0.4) | 13 (0.4) | 0.1 | 0.464 |
|  |  |  |  |  |
| *Sex^*^* |  |  |  |  |
| Male | 2792 (46.2) | 1493 (49.1) | 1.6 | 0.06 |
| Female | 3257 (53.8) | 1551 (51.0) | -1.5 | 0.07 |
|  |  |  |  |  |
| Total diagnoses made^€^ | 9721 | 5148 |  |  |
| *Diagnosis^%^* |  |  |  |  |
| Malaria | 4400 (45.3) | 1162 (23.3) | -27.2 | <0.001 |
| Respiratory tract infection | 2644 (27.2) | 1569 (30.5) | 4.2 | <0.001 |
| Pneumonia | 59 (0.6) | 72 (1.4) | 4.9 | <0.001 |
| Diarrhoea | 538 (5.5) | 361 (7.0) | 3.6 | <0.001 |
| Helminths | 428 (4.4) | 437 (8.5) | 10.1 | <0.001 |
| Other | 1652 (17.0) | 1547 (30.1) | 18.4 | <0.001 |
| Average number of diagnoses per visit | 1.6 | 1.7 |  |  |

**Table A: Characteristics and diagnoses made of children attending all three health centres.**

^$^Age missing 59 pre-intervention period, 36 intervention-period

*Sex missing 61 pre-intervention period and 39 intervention-period.

€ It was possible for children to have more than one diagnosis

^%^RTI includes cough, cold, flu, excludes pneumonia, TB and asthma.

Other diagnoses pre-intervention include: Skin infections (1.9%), burns, wounds, injuries (0.3%), eye infections (2.2%), epilepsy (0.2%), ear conditions (1.4%), gastro intestinal infections (0.6%), STIs (0.03%) fungal infections (0.2%), viral infections (0.07%)

Other diagnoses intervention-period include: Skin infections (3.9%), burns, wounds, injuries (1.0%), eye infections (9.0%), epilepsy (0.9%), ear conditions (2.8%), gastro intestinal infections (0.8%), STIs (0.1%), fungal infections (1.1%), and viral infections (1.0%)

**Table B:** Changes in level and trend of malaria visits at three health centres, results from a segmented linear regression model, unadjusted and adjusted results.

|  | **Malaria visits unadjusted** | | | | | **Malaria visits adjusted^^^** | | | | |
| --- | --- | --- | --- | --- | --- | --- | --- | --- | --- | --- |
|  | **Kikongi**  **Health Centre II** | | **Kikarara**  **Health Centre II** | | **Bwambara**  **Health Centre III^$^** | **Kikongi**  **Health Centre II** | | **Kikarara**  **Health Centre II** | | **Bwambara**  **Health Centre III** |
|  |  | |  | |  |  | |  | |  |
| Constant ($\beta_{0}$) | 33.9*** | | 8.3 | | 141.6*** | 33.1** | | 7.3 | | 147.5*** |
|  | (11.8) | | (16.5) | | (43.6) | (10.6) | | (16.4) | | (21.2) |
|  |  | |  | |  |  | |  | |  |
| Secular trend ($\beta_{1}$) | 9.4*** | | 12.7*** | | 1.7 | 8.1*** | | 12.0*** | | 3.5 |
|  | (1.6) | | (2.2) | | (5.3) | (1.5) | | (2.3) | | (3.0) |
|  |  | |  | |  |  | |  | |  |
| Change in level after intervention starts ($\beta_{2}$) | -126.8*** | | -125.7*** | | -44.2 | -118.4*** | | -121.2*** | | -129.2*** |
|  | (13.7) | | (19.8) | | (35.0) | (12.6) | | (20.1) | | (25.3) |
|  |  | |  | |  |  | |  | |  |
| Change in slope after intervention starts ($\beta_{3}$) | -9.5*** | | -14.1*** | | -6.4 | -8.5*** | | -13.5*** | | -5.3 |
|  | (1.8) | | (2.5) | | (6.8) | (1.6) | | (2.5) | | (3.2) |
|  |  | |  | |  |  | |  | |  |
| Rainy season ($\beta_{4}$) |  | |  | |  | 18.3** | | 11.5 | | 21.0 |
|  |  | |  | |  | (6.5) | | (10.2) | | (13.1) |
|  |  |  | |  | |  |  | |  | |

Standard errors in parenthesis *p<0.05, p<0.01**, p<0.001***; ^$^Model includes an adjustment for autocorrelation, ^adjusted for bimodal rainy seasons.

**Table C:** Percentage change at each health centre for malaria visits over time unadjusted and adjusted results.

|  | **Malaria visits decrease unadjusted** | | |  | **Malaria visits decrease adjusted**^^^ | | | |
| --- | --- | --- | --- | --- | --- | --- | --- | --- |
|  | **Kikongi Health Centre II** | **Kikarara Health Centre II^*^** | **Bwambara**  **Health Centre III^$*^** |  | | **Kikongi**  **Health Centre II** | **Kikarara**  **Health Centre II^*^** | **Bwambara Health Centre III^*^** |
| % Change 3 months after intervention starts | -89.4 | -89.4 | -69.7 |  | | -93.6 | -87.9 | -73.8 |
| % Change 6 months after intervention starts | -91.0 | -92.8 | -77.9 |  | | -95.0 | -91.7 | -77.5 |
| % Change 12 months after intervention starts | -93.0 | -97.1 | -90.4 |  | | -97.0 | -96.5 | -84.0 |
| % Change 18 months after intervention starts | -94.3 | -97.7 | -99.5 |  | | -98.3 | -99.5 | -89.4 |

^$^Model includes an adjustment for autocorrelation, *Percentage changes not significantly different from zero, ^adjusted for bimodal rainy seasons.

**Table D** Changes in level and trend of non-malaria visits at each health centre, results from a segmented linear regression model, unadjusted and adjusted results.

|  | **Non-malaria visits unadjusted** | | | **Non-malaria visits adjusted^^^** | | |
| --- | --- | --- | --- | --- | --- | --- |
|  | **Kikongi**  **Health Centre II** | **Kikarara**  **Health Centre II** | **Bwambara**  **Health Centre III^$^** | **Kikongi**  **Health Centre II** | **Kikarara**  **Health Centre II** | **Bwambara**  **Health Centre III** |
|  |  |  |  |  |  |  |
| Constant ($\beta_{0}$) | 40.7*** | -8.0 | 70.2*** | 41.4*** | -8.3 | 70.4*** |
|  | (10.2) | (14.3) | (10.8) | (9.5) | (14.9) | (11.0) |
|  |  |  |  |  |  |  |
| Secular trend ($\beta_{1}$) | -0.5 | 5.7** | 1.3 | -0.5 | 5.6** | 1.5 |
|  | (1.4) | (1.9) | (1.5) | (1.3) | (2.0) | (1.6) |
|  |  |  |  |  |  |  |
| Change in level after intervention starts ($\beta_{2}$) | 0.1 | -23.4 | -31.4 | 0.5 | -23.4 | -32.8* |
|  | (11.7) | (15.2) | (12.6) | (11.3) | (15.7) | (13.1) |
|  |  |  |  |  |  |  |
| Change in slope after intervention starts ($\beta_{3}$) | 2.0 | -6.3** | 2.3 | 2.0 | -6.2* | 2.1 |
|  | (1.5) | (2.2) | (1.6) | (1.5) | (2.3) | (1.7) |
|  |  |  |  |  |  |  |
| Rainy season ($\beta_{4}$) |  |  |  | -1.0 | 3.1 | -3.2 |
|  |  |  |  | (5.8) | (6.6) | (6.8) |
|  |  |  |  |  |  |  |

Standard errors in parenthesis *p<0.05, p<0.01**, p<0.001***; ^$^model includes an adjustment for autocorrelation, ^adjusted for bimodal rainy seasons.

**Table E:** Percentage change at each health centre for non-malaria visits over time unadjusted and adjusted results.

|  | **Non-malaria visits unadjusted^*^** | | |  | **Non-malaria visits adjusted^^*^** | | | | |
| --- | --- | --- | --- | --- | --- | --- | --- | --- | --- |
|  | **Kikongi Health Centre II^$^** | **Kikarara Health Centre II^$^** | **Bwambara**  **Health Centre III^$^** |  | | **Kikongi**  **Health Centre II** | **Kikarara**  **Health Centre II^$^** | **Bwambara Health Centre III** |  |
| % Change 3 months after intervention starts | +23.9 | -58.5 | -24.4 |  | | +26.3 | -59.6 | -25.7 |  |
| % Change 6 months after intervention starts | +43.5 | -67.4 | -16.2 |  | | +47.2 | -68.5 | -18.1 |  |
| % Change 12 months after intervention starts | +88.9 | -78.5 | -1.6 |  | | +96.1 | -79.6 | -5.0 |  |
| % Change 18 months after intervention starts | +144.5 | 85.1 | +10.9 |  | | +157.4 | -86.1 | +6.2 |  |

^$^Model includes an adjustment for autocorrelation, *percentage changes not significantly different from zero, ^adjusted for bimodal rainy seasons.

**Table F:** Changes in level and trend of overall visits at three health centres, results from a segmented linear regression model, unadjusted and adjusted results.

|  | **Overall visits unadjusted** | | | **Overall visits adjusted^^^** | | | |
| --- | --- | --- | --- | --- | --- | --- | --- |
|  | **Kikongi**  **Health Centre II** | **Kikarara**  **Health Centre II** | **Bwambara**  **Health Centre III^$^** | **Kikongi**  **Health Centre II** | **Kikarara**  **Health Centre II** | **Bwambara**  **Health Centre III** |  |
|  |  |  |  |  |  |  |  |
| Constant ($\beta_{0}$) | 73.4*** | 0.52 | 219.4*** | 72.7*** | -0.5 | 218.5*** |  |
|  | (11.1) | (20.5) | (30.5) | (10.4) | (20.6) | (30.4) |  |
|  |  |  |  |  |  |  |  |
| Secular trend ($\beta_{1}$) | 9.2*** | 18.0*** | 5.6 | 8.2*** | 17.2*** | 4.4 |  |
|  | (1.5) | (2.8) | (4.1) | (1.5) | (2.9) | (4.2) |  |
|  |  |  |  |  |  |  |  |
| Change in level after intervention starts ($\beta_{2}$) | -132.4*** | -142.4*** | -156.0*** | -125.1*** | -137.9*** | -148.1*** |  |
|  | (13.0) | (24.6) | (34.6) | (12.7) | (25.2) | (35.3) |  |
|  |  |  |  |  |  |  |  |
| Change in slope after intervention starts ($\beta_{3}$) | -7.6*** | -19.8*** | -4.0 | -6.8*** | -19.1*** | -3.0 |  |
|  | (1.7) | (3.1) | (4.6) | (1.6) | (3.1) | (4.7) |  |
|  |  |  |  |  |  |  |  |
| Rainy season ($\beta_{4}$) |  |  |  | 14.9* | 12.0 | 18.5 |  |
|  |  |  |  | (7.0) | (12.8) | (17.0) |  |
|  |  |  |  |  |  |  |  |

Standard errors in parenthesis; ***p<0.001, **p<0.01, *p<0.05; ^$^model includes an adjustment for autocorrelation, ^adjusted for bimodal rainy seasons.

**Table G:** Percentage change at each health centre for overall visits over time unadjusted and adjusted results.

|  | **Overall visits decrease unadjusted** | | | **Overall visits decrease adjusted^^^** | | |  |
| --- | --- | --- | --- | --- | --- | --- | --- |
|  | **Kikongi**  **Health Centre II^$^** | **Kikarara**  **Health Centre II^*^** | **Bwambara**  **Health Centre III^$*^** | **Kikongi**  **Health Centre II** | **Kikarara**  **Health Centre II^*^** | **Bwambara**  **Health Centre III^*$^** | |
| % Change 3 months after intervention starts | -73.5 | -76.9 | -55.6 | -69.7 | -77.9 | -67.8 | |
| % Change 6 months after intervention starts | -74.5 | -82.1 | -56.4 | -71.0 | -83.1 | -67.8 | |
| % Change 12 months after intervention starts | -75.8 | -88.9 | -57.7 | -73.0 | -89.8 | -67.8 | |
| % Change 18 months after intervention starts | -76.8 | -92.9 | -58.9 | -74.4 | -94.0 | -67.8 | |

*Percentage changes not significantly different from zero in these health centres, ^$^model includes an adjustment for autocorrelation, ^adjusted for bimodal rainy seasons.
